# Lithium lanthanum titanate perovskite as an anode for lithium ion batteries

**DOI:** 10.1038/s41467-020-17233-1

**Published:** 2020-07-13

**Authors:** Lu Zhang, Xiaohua Zhang, Guiying Tian, Qinghua Zhang, Michael Knapp, Helmut Ehrenberg, Gang Chen, Zexiang Shen, Guochun Yang, Lin Gu, Fei Du

**Affiliations:** 1 Key Laboratory of Physics and Technology for Advanced Batteries (Ministry of Education), State Key Laboratory of Superhard Materials, College of Physics, Jilin University, 130012 Changchun, China; 2 Center for Advanced Optoelectronic Functional Materials Research and Key Laboratory for UV Light-Emitting Materials and Technology of Ministry of Education, Northeast Normal University, 130024 Changchun, China; 3 College of Chemical Engineering and Material Science, Tianjin University of Science & Technology, 300457 Tianjin, China; 4 Institute for Applied Materials (IAM-ESS), Karlsruhe Institute of Technology (KIT), Hermann-von-Helmholtz-Platz 1, 76344 Eggenstein-Leopoldshafen, Germany; 5 Beijing National Laboratory for Condensed Matter Physics, Institute of Physics, Chinese Academy of Science, 100190 Beijing, China; 6 Division of Physics and Applied Physics, School of Physical and Mathematical Sciences, Nanyang Technological University, Singapore, 637616 Singapore

**Keywords:** Batteries, Energy storage, Batteries

## Abstract

Conventional lithium-ion batteries embrace graphite anodes which operate at potential as low as metallic lithium, subjected to poor rate capability and safety issues. Among possible alternatives, oxides based on titanium redox couple, such as spinel Li_4_Ti_5_O_12_, have received renewed attention. Here we further expand the horizon to include a perovskite structured titanate La_0.5_Li_0.5_TiO_3_ into this promising family of anode materials. With average potential of around 1.0 V vs. Li^+^/Li, this anode exhibits high specific capacity of 225 mA h g^−1^ and sustains 3000 cycles involving a reversible phase transition. Without decrease the particle size from micro to nano scale, its rate performance has exceeded the nanostructured Li_4_Ti_5_O_12_. Further characterizations and calculations reveal that pseudocapacitance dictates the lithium storage process and the favorable ion and electronic transport is responsible for the rate enhancement. Our findings provide fresh impetus to the identification and development of titanium-based anode materials with desired electrochemical properties.

## Abstract (web summary)

Exploration of high performance materials for lithium storage presents as a critical challenge. Here authors report micron-sized La_0.5_Li_0.5_TiO_3_ as a promising anode material, which demonstrates improved capacity, rate capability and suitable voltage as anode for lithium ion batteries.

## Introduction

Driven by the ever-growing needs for the plug-in electric vehicles (EVs) and smart grid, the development of lithium-ion batteries (LIBs) with high energy and power densities is more urgent than before^1–3^. To date, graphite and spinel Li_4_Ti_5_O_12_ are the most successful anode materials for LIBs, which have been widely used in the commercial LIBs^4,5^. Though graphite can deliver a high specific capacity of 372 mA h g^−1^, the low operation voltage near lithium plating is likely to raise the concern of battery safety^4^. Spinel Li_4_Ti_5_O_12_ is known as a high-rate anode material, whereas, the drawbacks, such as low capacity and over-high working potentials (1.55 V vs. Li^+^/Li), could strongly limit the output energy density on full-cell level^6^. Thus, there still remains a great challenge to exploit suitable host materials for Li^+^ with dual functions of high capacity and safe potential. Recently, Bruce et al. proposed a layered compound, Li(V_0.5_Ti_0.5_)S_2_, showing a low voltage of 0.9 V^7^. Though a high reversible capacity of 220 mA h g^−1^ was achieved at 0.9 C-rate, 99% of its theoretical one, Li(V_0.5_Ti_0.5_)S_2_ still showed unsatisfied rate capability and insufficient cycle life. Li_2_TiSiO_5_ is another interesting layered material that is composed with infinite sheets of distorted TiO_6_ octahedra and SiO_4_ tetrahedra, linked by Li atoms^8^. The anode exhibited a working potential of 0.28 V vs. Li^+^/Li and a specific capacity of nearly 200 mA h g^−1^ owing to the two-electron conversion reaction between TiO and Li_4_SiO_4_. Nevertheless, the conversion reaction is likely to induce irreversible phase separation and grain boundary movement with the result of critical misfit strain, particle cracking, and pulverization. Thus, optimization of appropriate crystal structure with controlled volume change could help to achieve better electrochemical reversibility and stable cycling.

Besides the high capacity and suitable voltage, the high-rate capability of a material is also essential for LIBs that can alleviate technological challenges associated with the adoption of EVs and grid-scale batteries^9^. Universally, maximum power output and minimum charging time of a rechargeable battery depend on both ionic and electronic transport^10,11^. And ionic diffusion within the active particles generally represents a fundamental limitation to the rate when being charged and discharged^12^. To date, the most commonly used strategy to improve the rate performance is to reduce the material dimension into nanometer, which can minimize the ionic diffusion distance^13^. However, nanocrystallization could be detrimental for volumetric packing density, manufacture cost, stability, and sustainability^14^. As an alternative approach, several bulk materials are reported to possess intercalation pseudocapacitive charge storage behavior that occur when ions intercalate into the tunnels or layers of the active materials accompanied by a faradaic charge transfer with no crystallographic phase transition^15–18^. The most notable feature provided by the intercalation pseudocapacitive reaction is the rapid charge storage and release processes that might offer a reasonable compromise between the capacity and high rate.

**Fig. 1:**
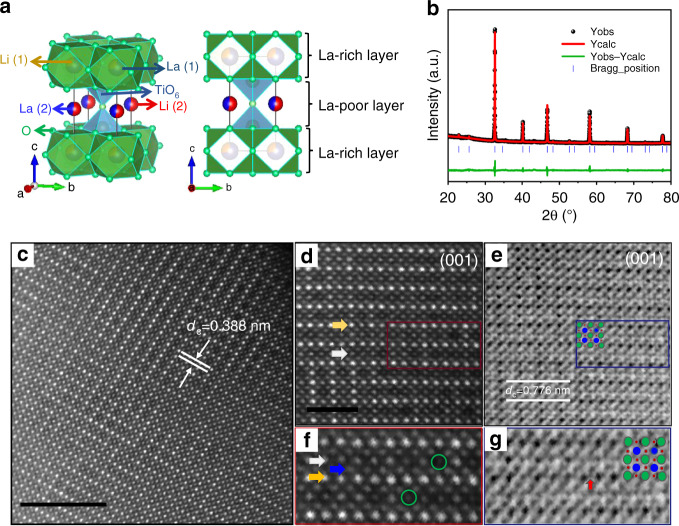
Structural properties of the as-prepared LLTO. **a** Schematic crystal structure; **b** Rietveld refinement based on powder XRD; **c** Large area HAADF images of LLTO perovskites along the [100] zone axis. Scale bar: 5 nm; **d** HAADF-STEM image of LLTO. Scale bar: 2 nm; **e** ABF-STEM image; **f** Magnified of HAADF-STEM; **g** Magnified of ABF-STEM. The green, blue and red balls in **e** and **g** represent the atoms of La, Ti and O, respectively.

Inspired by previous investigations about ionic conducting solids, such as NaTi_2_(PO_4_)_3_ in sodium-ion batteries^19^, here we explore the lithium ionic conducting structure to identify the appropriate host lattice that exhibits favorable Li diffusion properties as well as challenges the general criteria of particle dimension for the electrode material. La_2/3-s/3_Li_s_TiO_3_ is well known as superionic conductor with high ionic conductivity of *σ* ≈ 10^−3^ Ω^−1^ cm^−1^ at room temperature^20^. It crystallizes in the sturdy perovskite type structure made up of TiO_6_ octahedra framework stabilized by La atoms and have a large number of vacant sites at the unoccupied 18*d* and 6*a* positions, as displayed in Fig. 1a, which could participate in the Li storage and motion^21^. Furthermore, La_2/3-s/3_Li_s_TiO_3_ experiences an electronic transition from insulator to metal at high levels of Li insertion, corresponding to potentials below 1.5 V vs. Li^+^/Li. This qualifies La_2/3-s/3_Li_s_TiO_3_ as an ideal electrode candidate for high-rate LIBs^22^. Though the electrochemical properties of Li-poor perovskite (Li_0.27_La_0.54_TiO_3_) were reported once, the low reversible capacity (below 150 mA h g^−1^) still needs further effort to be improved, as well as the long-term stability and high-rate capability^23^.

Herein, we report the synthesis of the Li-rich compound, La_0.5_Li_0.5_TiO_3_, via gram-scale solid state reaction. Even crystallized in the micrometer-sized particles, the anode still demonstrates excellent lithium storage performance that exceeds those of nanostructured derivatives of heavily studied Li_4_Ti_5_O_12_^24^ and TiO_2_^25^. Combined with the lower lithiated potential (below 1.0 V), higher reversible capacity (above 200 mA h g^−1^) and superior high-rate capability, La_0.5_Li_0.5_TiO_3_ could be a promising alternative to the commercial Li_4_Ti_5_O_12_ as a high-performance anode material for LIBs.

## Results

### Crystal structure of La_0.5_Li_0.5_TiO_3_ and characterization

Figure 1b presents the Rietveld refinement of the X-ray diffraction pattern of as-prepared La_0.5_Li_0.5_TiO_3_ (LLTO). The structural analysis suggests that LLTO crystallizes into the tetragonal phase with space group P4/mmm. The lattice parameters are refined to *a* = *b* = 3.8811 Å and *c* = 7.7591 Å, consistent with the previous reported values^26^. As listed in Supplementary Table 1, there are two La positions in the refined structure: La-rich ones at 1*a* site and La-poor at 1*b* site, alternately stacked along the *c* axis. The unequal distribution of vacancy results in the doubling of the *c*-axis parameter and the appearance of a superstructure^20^.

To obtain more structural information of the pristine material, atomic-resolution annular bright-field (ABF) and high-angle annular dark-field (HAADF) STEM images recorded along the [100] zone axis are shown in Fig. 1c–g. The positions of La (*Z* = 57) and Ti (*Z* = 22) are clearly revealed by the bright dots in the HAADF-STEM images and dark dots in the ABF-STEM images. The distance of the nearest-neighbor layers HAADF image (Fig. 1c) is measured as ca. 0.388 nm, consistent with the interplanar spacing of (100) planes for the perovskite LLTO structure. As shown in the enlarged part of HAADF-STEM images along the [100] zone axis (Fig. 1d), there are two different La positions, which can be named as La-rich layer (yellow line) and La-poor layer (white line). Consistent with the results of Rietveld refinement, vacancies in the La-poor layer can be clearly observed (green circle in Fig. 1f). The bright dots in between La-rich and La-poor layers can be attributed to Ti atoms. The ABF-STEM observations of atomic positions at (100) planes are highly consistent with the perovskite ABO_3_ model, as shown in Fig. 1e,g for convenient visualization. Besides, O atoms can also be distinguished clearly between adjacent Ti atoms, as indicated by the red arrow. In addition, the distance of the nearest-neighbor La-rich layer is evaluated as 0.776 nm, matching well with the schematic figure of perovskite structure (Fig. 1a). The scanning electron microscope (SEM) image of LLTO (Supplementary Fig. 1) shows the bulk morphology with particle size ranging from several to dozens of microns owing to the high-temperature sintering process. Energy-dispersive spectroscopy mapping suggests a uniform distribution of La, Ti, and O in the as-prepared material as shown in Supplementary Fig. 2. The high-resolution X-ray photoelectron spectroscopy spectrum of Ti in LLTO in Supplementary Fig. 3 exhibits a double-peak feature with obvious asymmetry, whose binding energies are located at 458.2 and 463.6 eV, consistent with Ti^4+^ 2p_2/3_ and 2p_1/2_, respectively. After being deconvoluted, an additional peak can be observed at 456.4 eV, corresponding to Ti^3+^, owing to the partial loss of Li and La during the high-temperature sintering^27^.

### Lithium storage performance

**Fig. 2:**
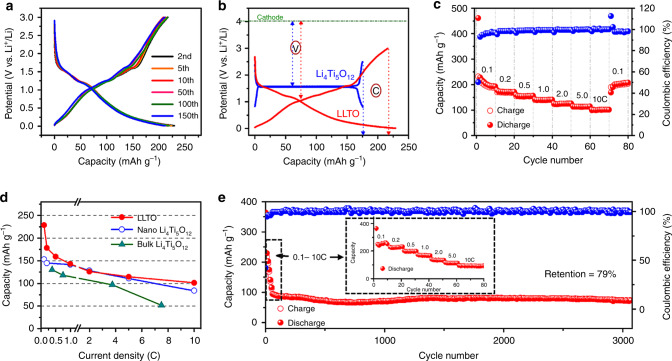
Electrochemical properties of LLTO. **a** Discharge–charge profiles since the second cycle at 0.1 C; **b** Comparison of discharge–charge profiles between LLTO and Li_4_Ti_5_O_12_; **c** Rate capability; **d** Comparison of rate capability for bulk LLTO, bulk Li_4_Ti_5_O_12_^24^ and nano Li_4_Ti_5_O_12_ electrode; **e** Long-term cycle stability at 10 C.

The electrochemical properties of perovskite LLTO was investigated by assembling the CR2032 coin-type cells with metallic lithium as the counter electrode. The anode delivers an initial discharge capacity of 449 mA h g^−1^ at the current density of 0.1 C-rate (1 C = 200 mA g^−1^) (Supplementary Fig. 4). Unfortunately, the charge capacity is restored to 229 mA h g^−1^. The relatively low Coulmbic effeciency (CE) might be mainly related to the decomposition of carbonate-based electrolyte (in our case: ethylene carbonate (EC)/dimethyl carbonate (DMC)/ethyl methyl carbonate (EMC) = 1:1:8) to form solid electrolyte interfacial (SEI) film below 0.5 V. To solve this issue, a common strategy is to introduce additives, such as VC^28^, K_2_CO_3_^29^, and etc. into the electrolyte to help to form a stable SEI and increase initial CE. Furthermore, the formation process usually used in commercial battery assembly can also be applied to eliminate the negative effect of SEI. Encouragingly, the CE increases to 94% and then stabilizes at nearly 100% (Supplementary Fig. 5). Furthermore, all the discharge–charge profiles are nearly superimposed on each other (Fig. 2a), demonstrating the excellent electrochemical reversibility. As compared with the discharge–charge profiles of spinel-Li_4_Ti_5_O_12_ (Fig. 2b), LLTO demonstrates dual merits of lower working potential and higher capacity, showing great potential in the commercial application. LLTO also delivers a higher reversible capacity than the Li-poor material La_0.56_Li_0.33_TiO_3_ (Supplementary Fig. 6). Besides, LLTO electrode demonstrates superior rate capability than Li_4_Ti_5_O_12_. As presented in Fig. 2c, the electrode delivers the reversible capacity of nearly 100 mA h g^−1^ at high applied current density of 10 C rate. When the current density returns back to 0.1 C after 70 cycles, the specific capacity is achieved as 190 mA h g^−1^, comparable to its initial five cycles. Furthermore, as compared in Fig. 2d and Supplementary Table 2, the high-rate capability of LLTO is obviously superior to the bulk-type Li_4_Ti_5_O_12_ whose particle size is in the micrometer scale^24^, and even comparable to the commercial nano Li_4_Ti_5_O_12_ with particle size in 100–500 nm (SEM image in Supplementary Fig. 7). The outstanding rate performance of LLTO can be attributed to the intrinsic pseudocapacitance, which will be further discussed in the following section. In addition, the long-term stability was examined after the current densities progressively increased to 10 C, which delivers the capacity retention of 79% after 3000 cycles (as compared with the starting scan at 10 C rate) and a CE of nearly 100% (Fig. 2e).

### Kinetics analysis

**Fig. 3:**
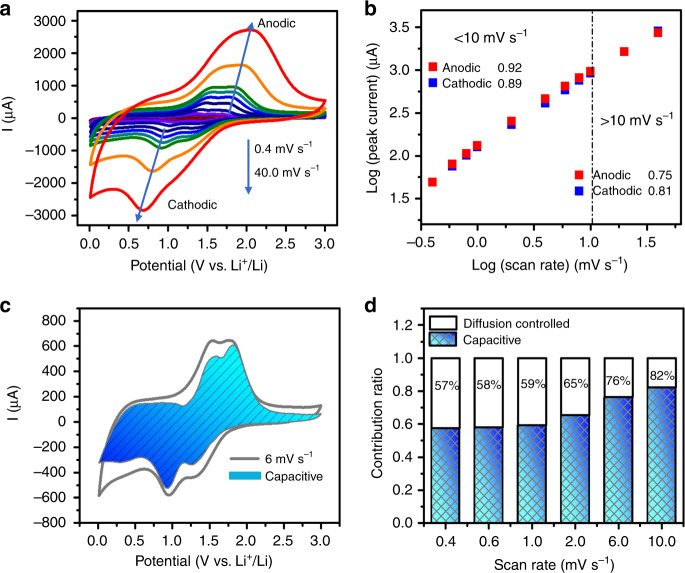
Kinetic properties of LLTO. **a** CV curves at different scanning rates; **b** Corresponding peak current versus square root of scan rates; **c** Separation of the capacitive and diffusion currents at a scan rate of 6 mV s^−1^; **d** The percentage of pseudocapacitive contribution at different scan rates.

Unlike other anode materials with micro size that usually suffer from the sluggish kinetic property, the micron-sized LLTO anode presents unexpectedly superior rate performance. To gain further insight into the origin of the outstanding rate performance, kinetics analysis based on cyclic voltammetry (CV) analysis was carried out. The CV curves for LLTO anode at different sweep rates from 0.4 to 40 mV s^−1^ (Fig. 3a) exhibit broad cathodic and anodic peaks with quasirectangular shapes stretching at all scan rates, which indicates a pseudocapacitivity contribution during lithiation and delithiation processes. According to the relationship between the response current (*i*) under a certain potential (V) and the sweep rate (*v*)^15,17^:

1 $$i_{(V)} = av^b,$$

2 $${\mathrm{log}}\,i_{(V)} = b\,{\mathrm{log}}\,v + {\mathrm{log}}\,a.$$

where the *b*-value is determined by the ion storage mechanism and can be calculated by slope of the log(*i*) − log(*v*) plot. To be more specific, the *b*-value of 0.5 means a completely diffusion-controlled process, whereas a *b*-value of 1.0 indicates a faradaic contribution from charge transfer with surface or subsurface atoms (pseudocapacitance effect) or the non-faradaic contribution from electrical double-layer effect^16^. As demonstrated in Fig. 3b, the *b*-values of 0.92 and 0.89 are calculated for the anodic and cathodic peaks, respectively, suggestive of a fast capacitive-controlled kinetics. A distinct inflection point corresponding to the decrease in the *b*-values of 0.75 (anodic peak) and 0.81 (cathodic peak) at sweep rates >10 mV s^−1^ is found, indicative of the limitation to the rate capability. This kind of transition has previously found in other materials with pseudocapacitive nature, such as T-Nb_2_O_5_^15^ and G-TiO_2_-B^17^, which may be caused by the enhanced ohmic resistance or diffusion constraints. Moreover, the relationship between capacity (*Q*) and (sweep rate)^−1/2^ (*v*^−1/2^) simulates this rate-limiting step of Li^+^ storage mechanism again, displayed in Supplementary Fig. 8. The *Q* plays an independent role with the change of sweep rate in the range of 0.4 and 10 mV s^−1^, which indicates the capacitive contributions. On the contrary, the linear decrease of *Q* upon the increase of *v*^−1/2^ in the region of *v* > 10 mV s^−1^ reflects the rate-limiting diffusion-controlled charge storage. Quantificationally, the capacitive contribution can be further confirmed by separating the response current (*i*) under a certain potential (V) into capacitive (linear relationship with sweep rate (*v*), marked as *k*_*1*(V)_*v*) and diffusion-controlled reactions (proportional to the (weep rate)^1/2^) (*v*^1/2^), represented by *k*_*2*(V)_*v*^1/2^), as the following equation^16^:

3 $$i_{({\mathrm{V}})} = k_{1({\mathrm{V}})}v + k_{2({\mathrm{V}})}v^{1/2},$$

4 $$i_{\left( {\mathrm{V}} \right)}/v^{1/2} = k_{1({\mathrm{V}})}v^{1/2} + k_2,$$

where both *k*_1_ and *k*_2_ are constants for a certain potential (V), and can be calculated by plotting *i*_(V)_/*v*^1/2^ versus *v*^1/2^, where *k*_1_ is the slope and *k*_2_ is the *Y*-intercept. Based on this method, a high dominant capacitive contribution of ca. 76% of the total charge at sweep rate of 6 mV s^−1^ (Fig. 3c) is obtained in the bulk LLTO anode. As the sweep rate increases, the role of capacitive contribution further increases (Fig. 3d) with a maximum value of ~82% at 10 mV s^−1^. In view of the above analysis, a pseudocapacitive charge storage mechanism could be established for LLTO that enables high-rate energy storage. Different from most of the pseudocapacitive transition metal oxides^17^ that depends on the particle size and surface area, LLTO demonstrates a strong pesudocapacitive contrbuiton even crystallizing into micron scale with limited surface areas that strongly suggests LLTO belongs to the type of instrinsic pseudocapacitance^30^. This phenomenon can be understood in terms of fast ionic and electronic conductivities, since the discharge/charge processes are double injection/release procedure of both ions and electrons^31^. According to the galvanostatic intermittent titration technique measurement (Supplementary Fig. 9 and Supplementary note 1), the lithium diffusion coefficient of LLTO during a typical lithiation process ranges from 10^−10^ to 10^−11^ cm^2^ s^−1^, which is two magnitude higher than that of Li_4_Ti_5_O_12_^6^, indicating a super fast kinetic property of Li^+^ ions. Furthermore, the subsequent first-principle calculations reveals a narrower band gap as compared with Li_4_Ti_5_O_12_ and an enhanced electronic conductivity for the lithiated LLTO due to the weak binding electron ability of Ti^3+^, and thus accelates the electrochemical raction. Since the exposure of surface to the electrolyte is not critical for the high-rate behavior, such instrinsic pseudocapacitive materails (e.g., LLTO), shoud be beneficial for the practical applications^30^.

## Discussion

**Fig. 4:**
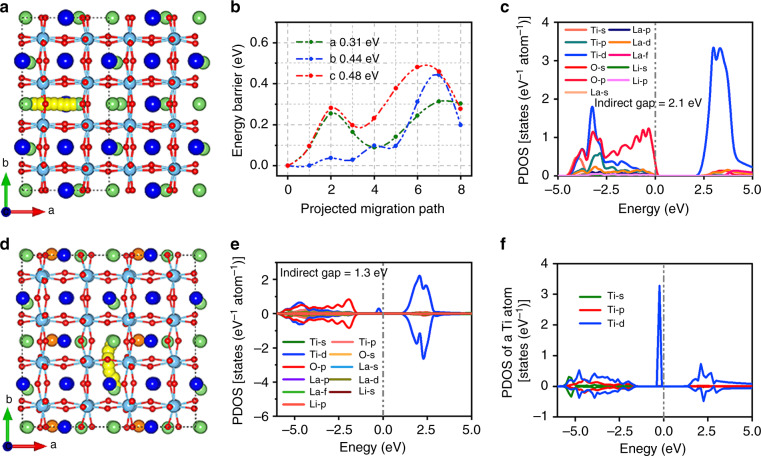
Geometric/electronic structures of LLTO and lithiated LLTO. **a** Crystal structure and the Li^+^ diffusion path with the lowest barrier; **b** Diffusion energy barrier at three-dimensional directions; **c** Projected density of states (DOS) of pristine LLTO; **d** Crystal structure with the Li^+^ diffusion path of lithiated LLTO; **e** PDOS of lithated LLTO; **f** PDOS of magnetized Ti atom adjacent to the inserted Li atom in lithium LLTO. The green, blue, cyan, and red spheres in crystal structure represent lithium, lanthanum, titanium, and oxygen atoms, respectively.

To gain more insight into the superior rate capability of LLTO, density functional theory (DFT)-based first-principle calculations were employed to analyze the ionic and electronic conductivity. Firstly, nudged elastic band calculations were applied to investigate Li^+^ diffusion mobility in La_0.5_Li_0.5_TiO_3_ along different directions, as displayed in Fig. 4a and Supplementary Fig. 10. The resultant low energy barrier of 0.31 eV, well consistent with the reported experimental value (0.29 eV)^26^, suggests a preferential diffusion pathway of Li^+^ ions along *a*-axis direction. Interestingly, all the energy barrier values along the *a*-axis (0.31 eV), *b*-axis (0.44 eV), and *c*-axis (0.48 eV), as shown in Fig. 4b, are much lower than those of Li_4_Ti_5_O_12_ anode^32,33^, not only hinting a superior capability of Li^+^ diffusion than Li_4_Ti_5_O_12_, but also implying a 3D diffusion path in the perovskite framework. Secondly, the projected density of states of LLTO is also calculated since the electronic conductivity is inversely proportional to the band gap. In another word, small band gap is conductive to the generation of intrinsic electrons or holes^34^. As exhibited in Fig. 4c, the band gap of LLTO is calculated as 2.1 eV which is much narrower than that of Li_4_Ti_5_O_12_ (3.8 eV)^35^. This phenomenon suggests LLTO can provide much faster electron compensation than Li_4_Ti_5_O_12_ during fast Li^+^ (de)intercalation. Moreover, the energy barrier and band gap of LLTO upon discharging are comprehensively investigated. Generally, Li^+^ ion prefers to be situated at the O_4_ window position^36^. Considering the spatial configuration, there are three possible positions (i.e., A_1_, A_2_, and A_3_) to accommodate Li^+^ ion, which are at the middle-point of La and La, Li, and La, and Li and Li, respectively (Supplementary Fig. 11). In comparison with the other two positions, Li^+^ ions are in favor of situating at A_3_ site owing to the lowest formation energy of Li interstitial (−1.47 eV in Supplementary Table 3)^37^. The corresponding crystal structure after Li^+^ insertion was visualized in Fig. 4d. After incorporation of Li^+^ ions, LLTO becomes magnetic and demonstrates smaller band gap (1.3 eV) as compared with the pristine one (Fig. 4e). In detail, the lithiation induces the reduction of adjacent Ti from +4 to +3 state, corresponding to the electron occupation from 3d^0^4s^0^ to 3d^1^4s^0^, whereas the oxidation state of La remains unchanged^22^. In octahedral crystal field, the d electron of transition metal prefers to occupy the *d*_xy_ orbit and maintains a high spin state^38^. Thus, the magnetism of lithiated LLTO (Fig. 4f) mainly originates from the reduction of Ti^4+^ with the octahedral configuations. Similar observation appears in lithiated Li_4_Ti_5_O_12_^39^. On the other hand, the spin-up Ti^3+^ states, sitting on the left side of the Fermi Level (Fig. 4f), are able to enter into the conduction band upon exciation with a small amount of energy and act as free electrons^20,40^. This can be attributed to the weak binding electron ability of Ti^3+^. Thus, the lithiated LLTO demonstrates good electronic conductivity. The calculated Li ion diffusion energy barrier upon discharging becomes slightly higher than the pristine ones, as shown in Supplementary Fig. 12. Similar phenomenon was also observed in other materials, possibly owing to the decrease of vacant Li sites^41^. Thus, the intrinsic low Li ion diffusion barrier and excellent conductivity of perovskite LLTO enables the ultrafast Li^+^ transportation.

**Fig. 5:**
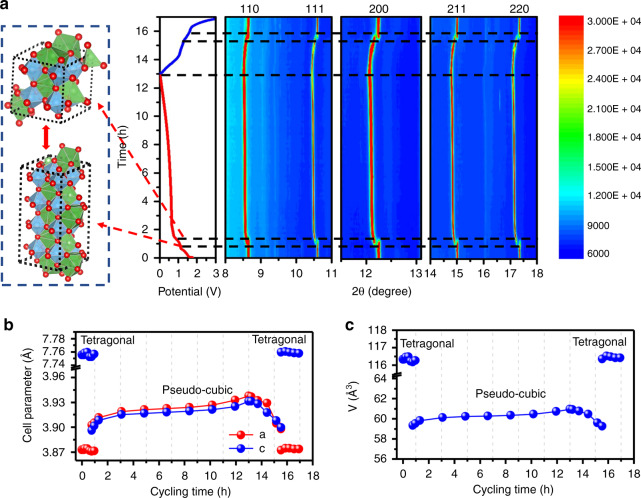
Structural transition upon Li^+^ insertion and extraction. **a** Contour maps of in situ synchrotron X-ray diffraction collected during the first charge–discharge with schematic structures; **b** Variation of lattice constants (*a, c*) and **c** unit cell volume (*V*) during the cycling process.

In addition, in situ synchrotron diffraction patterns for the initial charge–discharge processes were recorded to elucidate the structural evolution during the reversible Li^+^ insertion and extraction. Contour maps in the selected 2-theta ranges are shown in Fig. 5a together with the refined cell paremeters in Fig. 5b,c (represented Rietveld refiment patterns are given in Supplementary Figs. 13–15). At the open circuit voltage (OCV) stage, all the reflections of LLTO on a live cell are consistent with those of the pristine material, belonging to the tetragonal cell with space group of P4/mmm (Supplementary Fig. 13). When initially discharged to 1.2 V, all the reflections of LLTO remain nearly unchanged in both peak position and symmetry, as displayed in Supplementary Fig. 16, suggestive of nearly unchanged cell parameters (Fig. 5b). This interesting phenomenon can be understood in terms of the insertion of Li^+^ into the vacancy sites of perovskite LLTO. Further Li^+^ intercalation into the lattice induces gradual vanish of original LLTO reflections and appearance of several new ones within a narrow voltage region between 1.2 and 0.9 V, characteristic of a two-phase transition process. Interestingly, all the new reflections are located at their lower-angle sides with no formation of additional peaks. A careful Rietveld refinement based on the synchrotron profile at discharged to 0.91 V (Supplementary Fig. 15) suggests a pseudo-cubic symmetry, whose *c* value is only half of its initial tetragonal structure (Fig. 5b, c). In the pristine structure, the ordered arrangement of La-rich and La-poor layers induces a superstructure with doubled unit cell along the *c* axis^36^. Upon Li^+^ insertion, the vacancies are filled and both sites become equivalent, which results in the disappearance of superstructure and formation of cubic unit cell. A similar transition was once observed in the quenched perovskite Li_0.3_La_0.567_TiO_3_ materials owing to the correlated local ordering of La vacancies and occupied Li sites^42^. When further discharged to 0.01 V, no additional diffraction peaks beyond pseudo-cubic LLTO phase can be detected. And all reflections shift toward the low-angle side, indicative of a solid-solution reaction. The long sloping discharge curve and corresponding long scanning time are related to the formation of SEI film that could be further improved via optimization of electroyte component or utilization of formation process before application. In the charging processes, all the reflections of pseudo-cubic LLTO shifts to their high-angle side, consistent with the lattice shrinkage process after Li^+^ extraction. When charging to 1.2 V, a visible biphasic region can be observed with nearly same voltage variation (0.3 V) in the discharging process. Above 1.5 V, the biphasic transition finishes and all the reflections return back to their original positions at OCV sate. These results propose a highly reversible phase transition: a delithiated induced solid-solution accompanied by a two-phase transition from pseudo-cubic into tetragonal symmetry. As hinted by Fig. 5c, after one cycle, the volume expansion is calculated as small as 0.068%, which well explains the excellent electrochemical reversibility^5^. In addition, XRD diffractions of LLTO before and after 150 cycles were recorded and compared in Supplementary Fig. 17. There is no signs of new reflections or widening of these peaks, indicative of an excellent structural reversibility upon Li^+^ insertion and extraction.

In sum, perovskite-type La_0.5_Li_0.5_TiO_3_ was proposed as a low-potential intercalation-type anode for LIBs with a low working voltage below 1.0 V and reversible capacity of 225 mA h g^−1^. Even crystallized in the micrometer scale, the anode still demonstrated superior capability with nearly 100 mA h g^−1^ at 10 C-rate and stable cycle performance whose capacity retention is 79% over 3000 cycles. DFT calculations suggested a lower diffusion barrier and narrower band gap than the commercial used Li_4_Ti_5_O_12_, which enables La_0.5_Li_0.5_TiO_3_ as a promising low-voltage anode for practical application.

## Methods

### Synthesis of LLTO

In a typical synthesis, 0.012 mol Li_2_CO_3_ (An excess of 20 mol% Li_2_CO_3_ was added to compensate for Li loss during processing), 0.01 mol La_2_O_3_, and 0.04 mol TiO_2_, were mixed and ball-milled with ethyl alcohol in planetary ball mill machine at 300 rpm for 4 h. The mixture was dried at 120 °C for 12 h in the vacuum oven. Afterwards, the precursor was calcined at 800 °C for 8 h and 1250 °C for 12 h under ambient atmosphere. La_0.56_ Li_0.33_TiO_3_ was synthesized with the same method and different ratios.

### Physical characterization

The morphologies and the microstructures of the samples were investigated using the field emission scanning electron microscope (FESEM, JEOL JSM-6700F). X-ray diffraction (XRD) characterizations were conducted by a Rigaku D/max-2550 diffractometer with Cu-Kα radiation, and Rietveld refinement was carried out for the crystal structure analysis. Atomic-resolution ABF STEM and HAADF STEM were performed using a JEM ARM200CF (JEOL, Tokyo, Japan) transmission electron microscope equipped with double CEOS (CEOS, Heidelberg, Germany) probe aberration correctors. The attainable spatial resolution of the microscope is 78 pm. The valence states of the samples were identified by X-ray photoemission spectra by using a VG scientific ESCALAB-250 spectrometer.

### In situ synchrotron radiation diffraction

To monitor phase evolution of the LLTO electrode during the initial cycling process, in situ *synchrotron radiation diffraction* (HT-SRD) was performed at MSPD in ALBA using monochromatic synchrotron diffraction (*λ* = 0.413206 Å). Here, LaB_6_ were used as a standard sample for the calibration of the wavelength. Coin cells with glass windows (Li|LP30|LLTO) were assembled. During the in situ test, the cells were cycled at 40 mA g^−1^ in the potential range of 0.01–3.0 V vs. Li/Li^+^ at room temperature, equipped with a self-made electrochemical test cell setup^43^.

### DFT calculations

Structural optimization and electronic property calculations were carried out within density functional theory (DFT)^44^ as implemented in the Vienna Ab initio Simulation Package. Considering both of accuracy and computational efficiency, the Perdew–Burke–Ernzerhof generalized gradient approximation (GGA) exchange and correlation functional was used as compromise. The scalar relativistic projector augmented wave pseudopotentials were adopted to describe electron-ion interaction^45^. A plane-wave cutoff energy of 520 eV and a 2 × 2 × 2 k-point mesh was adopted. To treat the strong correlation effect, we used the GGA + U method with Ud = 5 eV and Jd = 0.64 eV for the Ti d states and Uf = 11 eV and Jf = 0.68 eV for La f states^46^. For pristine La_0.5_Li_0.5_TiO_3_ containing Li vacancy (VLi-LLTO), the structure was adopted from the retrieved fitted tetragonal phase with space group of P4/mmm. The energetically favorable site of Li vacancy was determined by calculating the defect formation energy of various Li positions in La_0.5_Li_0.5_TiO_3_. For cubic La_0.5_Li_0.5_TiO_3_ (LLTO), the structure was adopted from the retrieved fitted pseudo-cubic structure. Same as above, a supercell of 2 × 2 × 2 was constructed. For LLTO after lithiated (LLTO + Li), there are three possible positions to accommodate Li atoms, which are at the middle-point of Li and La, Li and Li, and La and La, respectively. The formation energy of Li interstitial was calculated as follows^36^:

5 $$E = E({\mathrm{LLTO + Li}})\;-\;E({\mathrm{LLTO}})\;-\;E({\mathrm{Li}}),$$

where the *E*(LLTO + Li) and *E*(LLTO) are the total energy of LLTO + Li and LLTO, respectively. *E*(Li) is the energy of per atom in bulk Li of bcc phase.

The nudged elastic band method was used to calculate the energy barriers of Li ion migration. A suitable supercell was made to contain the pathway in the inner of crystal structure. In detail, for VLi-LLTO, a new supercell of 1 × 2 × 1 and 2 × 1 × 1 were constructed for the pathways along a and b directions, respectively. For LLTO + Li, a new supercell of 2 × 2 × 1 were constructed for the pathways along *b* and *c* directions.

### Electrochemical measurement

For fabrication of electrodes, active materials, Ketjen black conductive and carboxyl methyl cellulose binders were mixed in a weight ratio of 8:1:1 and dissolved in DI water to prepared the slurry casting on Cu foil current collector. These electrode films were dried in a vacuum oven at 60 °C for 10 h. The mass loading of electrodes was 1.5–2 mg cm^−2^. The electrochemical properties of the as-prepared electrodes were evaluated by assembling 2032-type coin cells in the argon filled glovebox. A Celgard 2320 membrane was employed as the separator, while lithium foils serve as the counter electrodes. The electrolyte was 1 M LiPF_6_ dissolved in a solvent of EC/DMC/EMC (1:1:8 v/v/v). Galvanostatic charge–discharge cycling was then performed on a Land-2001A (Wuhan, China) automatic battery tester, where 1 C corresponds to 200 mA g^−1^. CV was performed on a VSP multichannel potentiostatic-galvanostatic system (Bio-Logic SAS, France).

## Supplementary information

Supplementary Information

## Data Availability

The data that support the findings of this study are available from the corresponding author upon reasonable request.
