# Supplementary material for: Lithium lanthanum titanate perovskite as an anode for lithium ion batteries
Source: Nat Commun. 2020 Jul 13;11:3490. doi: 10.1038/s41467-020-17233-1 (PMC7359355; doi:10.1038/s41467-020-17233-1)
Supplement: Supplementary file 1 — Supplementary Information [file 41467_2020_17233_MOESM1_ESM.pdf]

Supplementary Information

Lithium lanthanum titanate perovskite as an anode for lithium ion batteries

Zhang et al.

Supplementary Figures

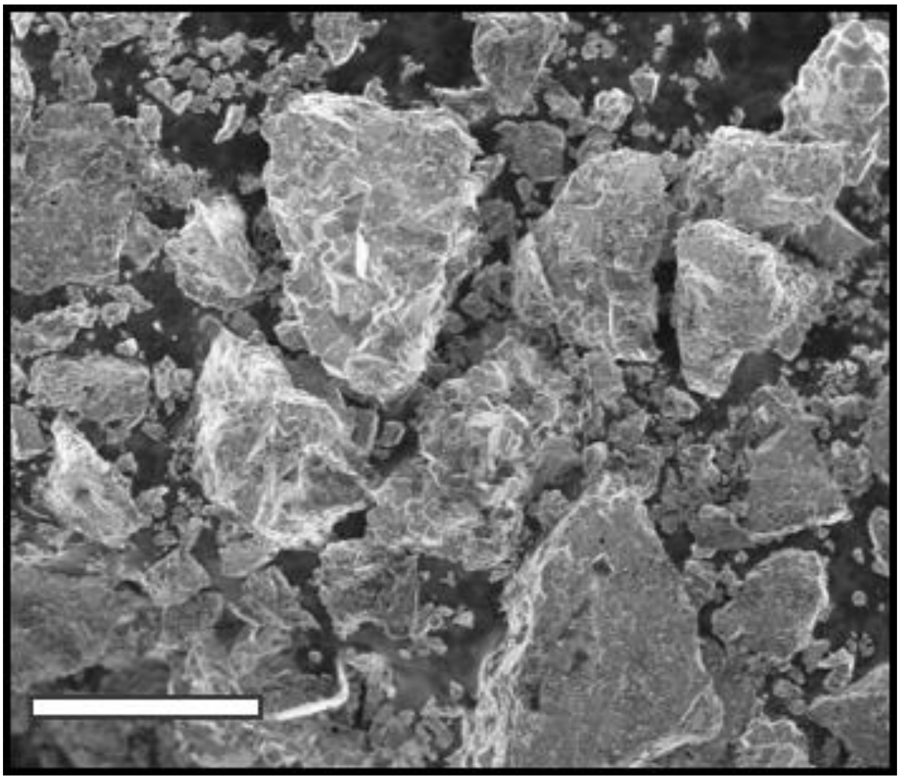

Supplementary Figure 1. SEM image of LLTO. Scale bar: 20 μm.

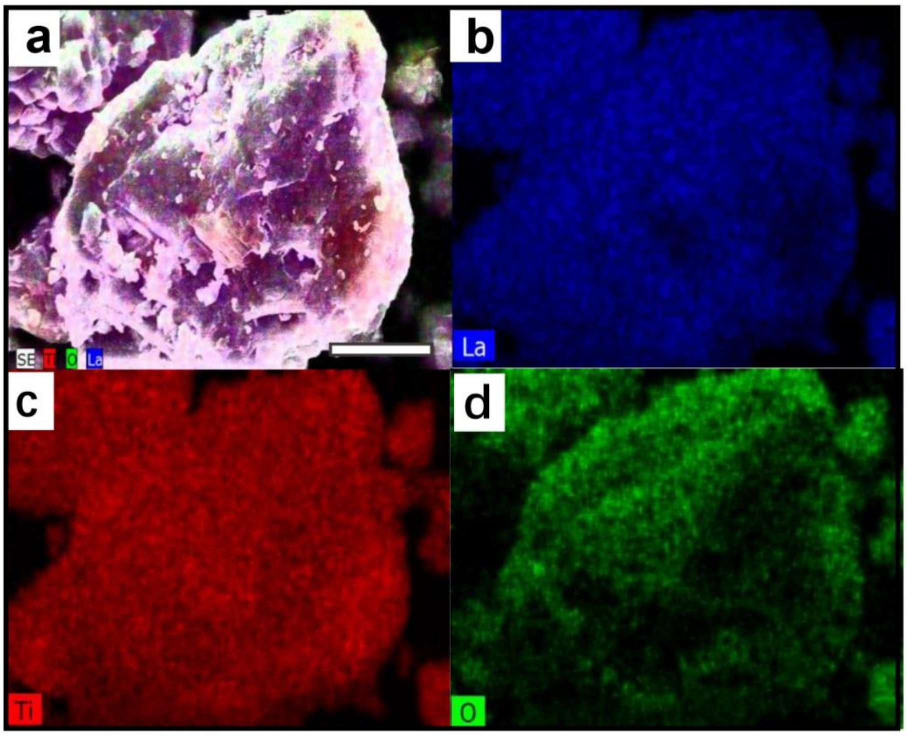

Supplementary Figure 2. Elemental analysis of LLTO. **(a)** SEM image and the corresponding Energy-dispersive spectroscopy (EDS) mapping of La**(b)**, Ti**(c)**, O**(d)**. Scale bar: 5 μm.

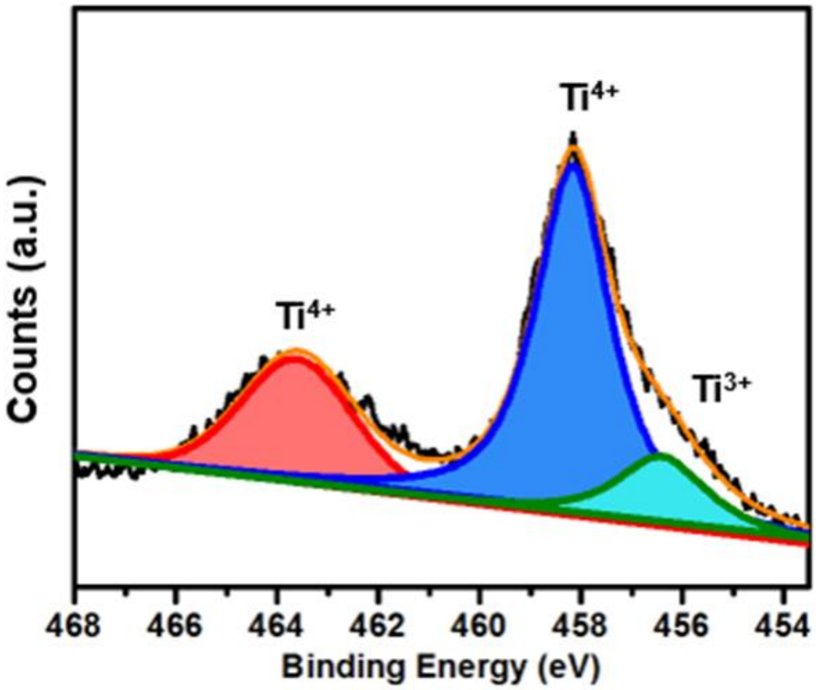

Supplementary Figure 3. High-resolution X-ray photoelectron spectroscopy spectrum of Ti in LLTO.

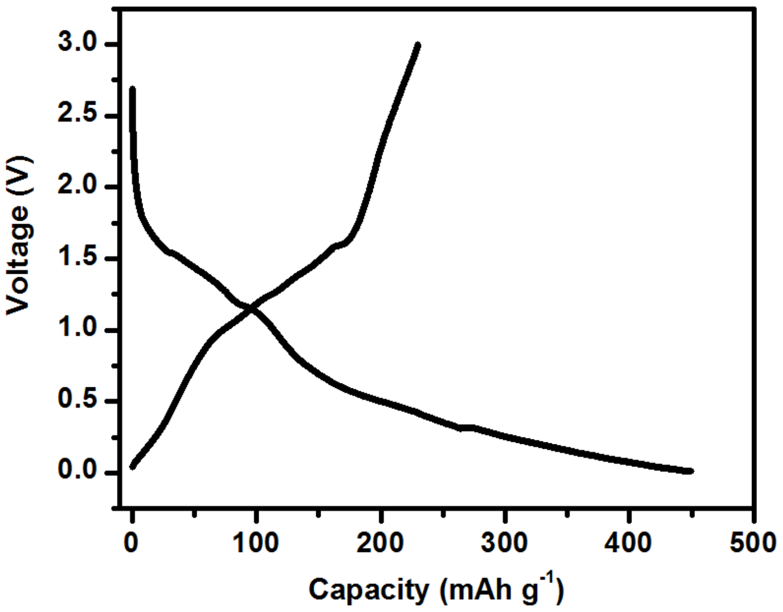

Supplementary Figure 4. The initial discharge-charge profile of LLTO.

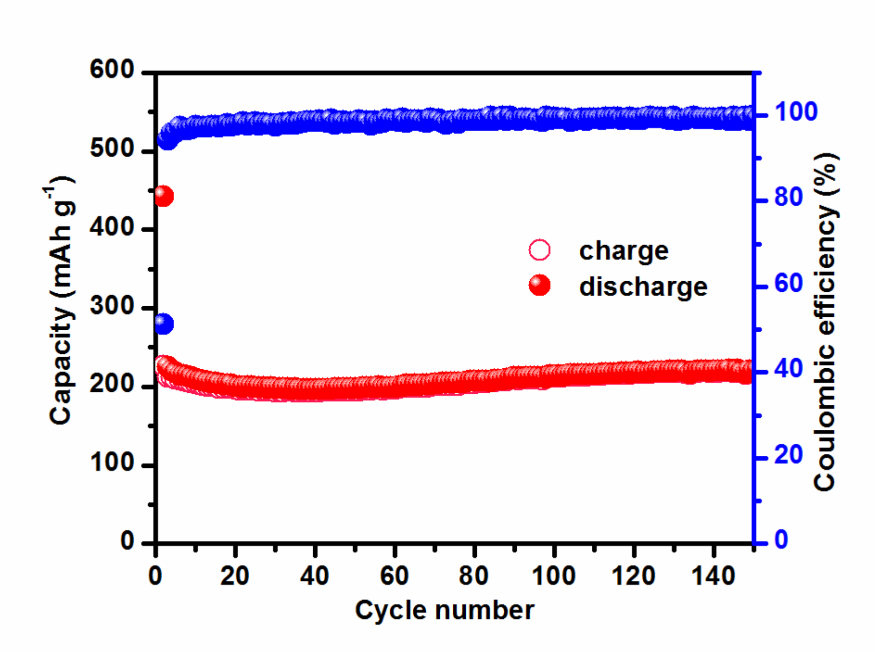

Supplementary Figure 5. Capacity versus cycle number at a current density of 0.1C of LLTO.

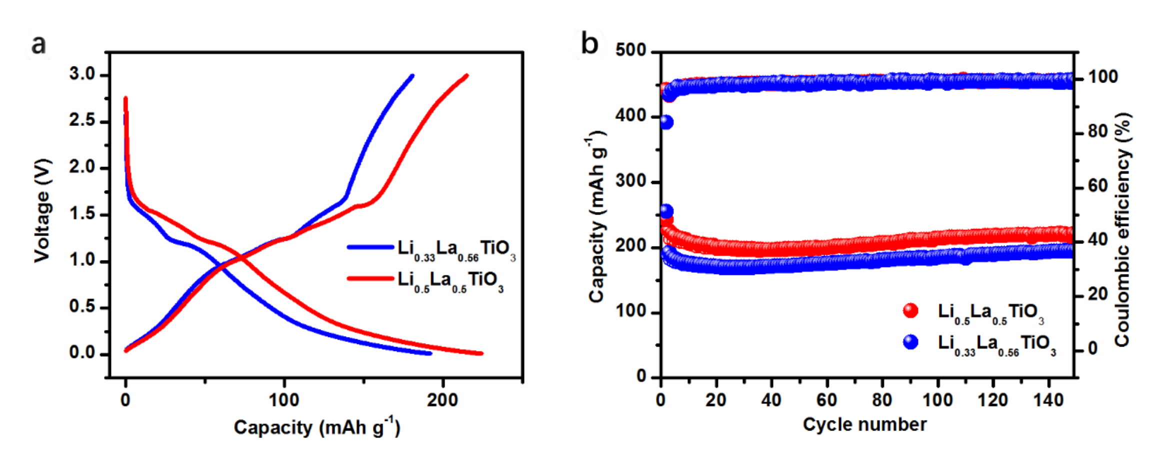

Supplementary Figure 6. The Li^+ storage performance of $Li_{0.5}La_{0.5}TiO_3$ and $Li_{0.33}La_{0.56}TiO_3$. **(a)** The second discharge-charge profiles; **(b)** capacity versus cycle number at 0.1C.

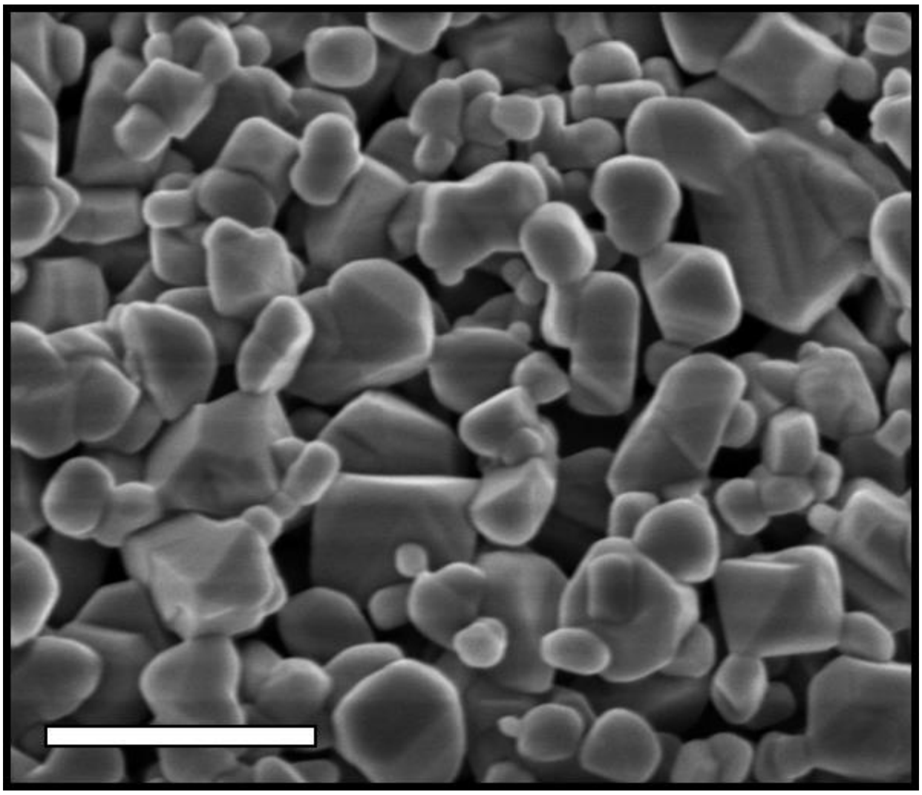

Supplementary Figure 7. SEM image of commercial nano $Li_4Ti_5O_{12}$. Scale bar: 1 μm.

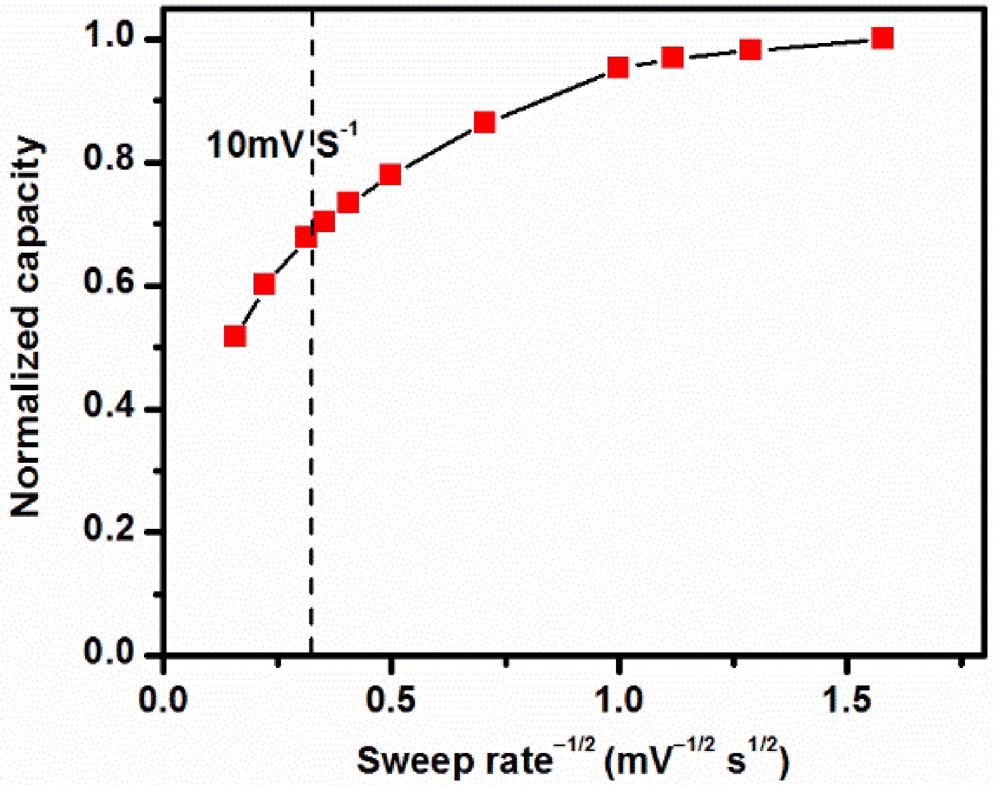

Supplementary Figure 8. Capacity versus scan rate$^{-1/2}$ of 0.4 – 40 mV s^{-1}.

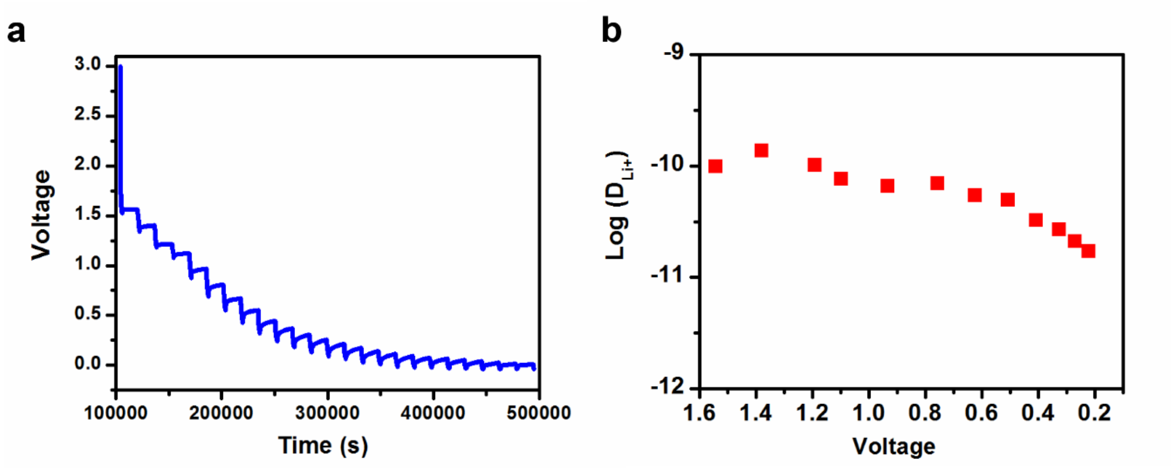

Supplementary Figure 9 The lithium diffusion coefficient of LLTO. **(a)** GITT curves for LLTO and **(b)** the lithium diffusion coefficient throughout the insertion process.

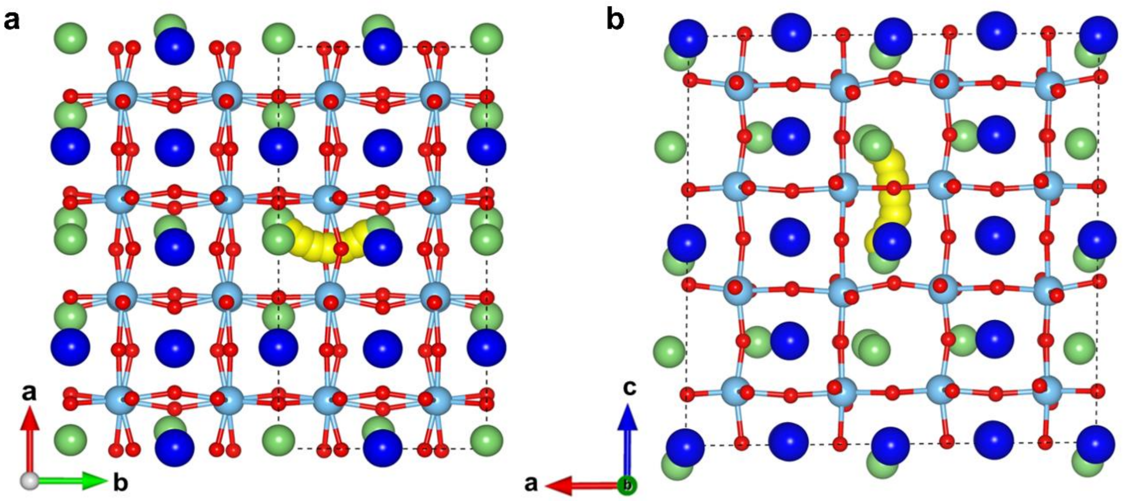

Supplementary Figure 10. The nudged elastic band (NEB) calculations result of Li^{+} diffusion road. **(a)** along b axis and (b) along c axis.

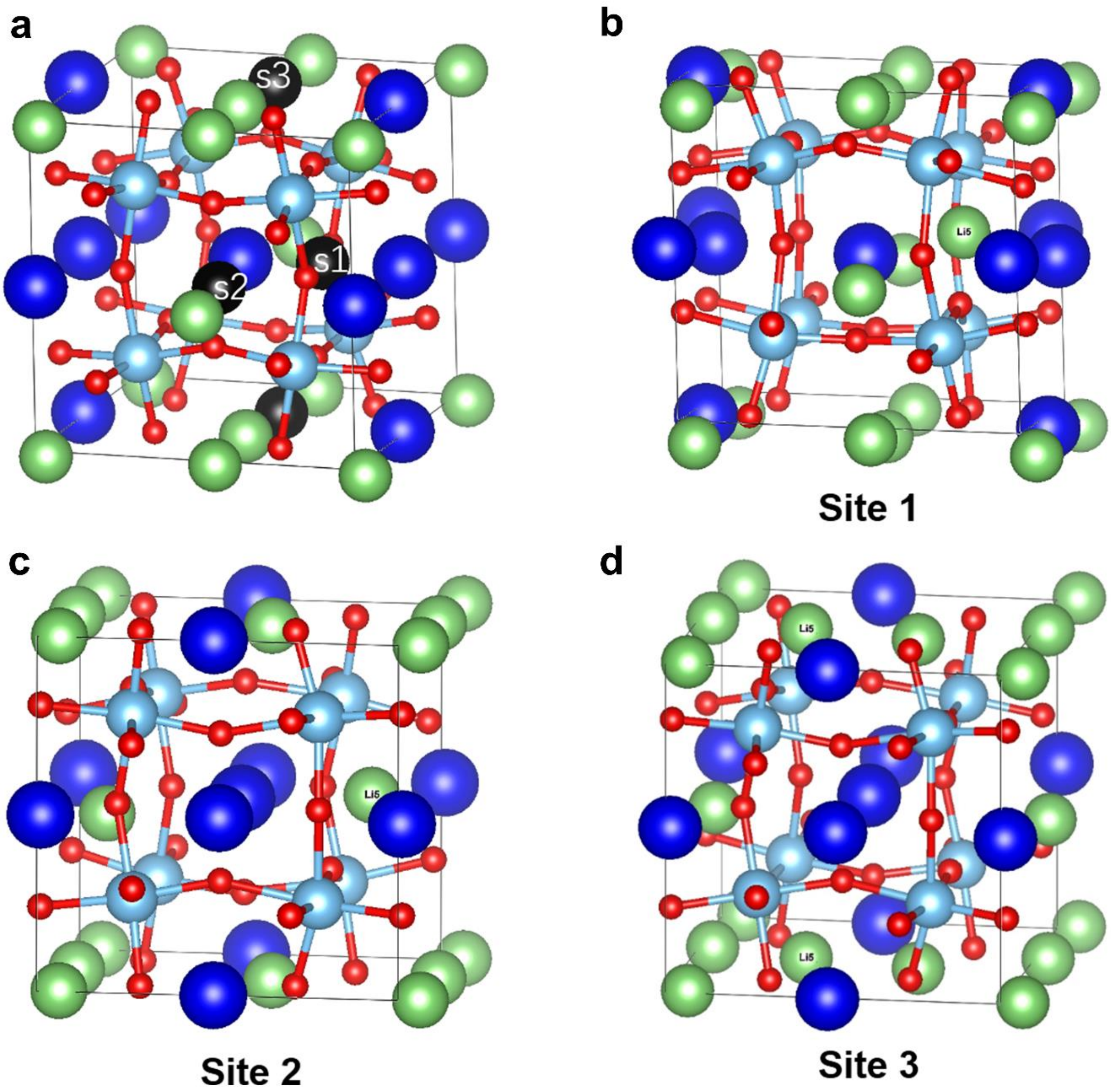

Supplementary Figure 11. DFT calculated LLTO structures after Li insertion. Three types of Li sites are expected in the structure**(a)**; **(b)** O_4 square window positions between La-La atoms (A_1); **(c)** O_4 square window positions between La-Li atoms (A_2); **(d)** O_4 square window positions between Li-Li atoms (A_3). The green, blue, cyan and red spheres represent lithium, lanthanum, titanium and oxygen atoms, respectively.

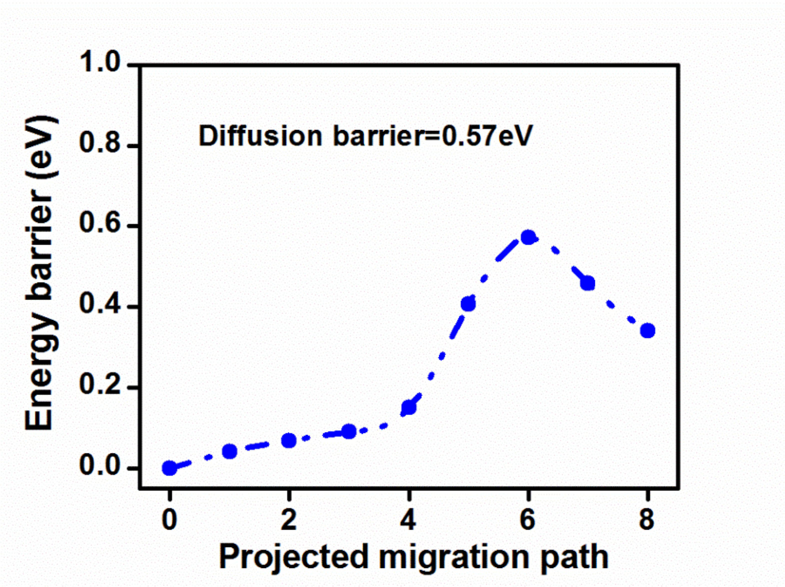

Supplementary Figure 12. The lowest Li ion diffusion energy barrier of LLTO after Li insertion.

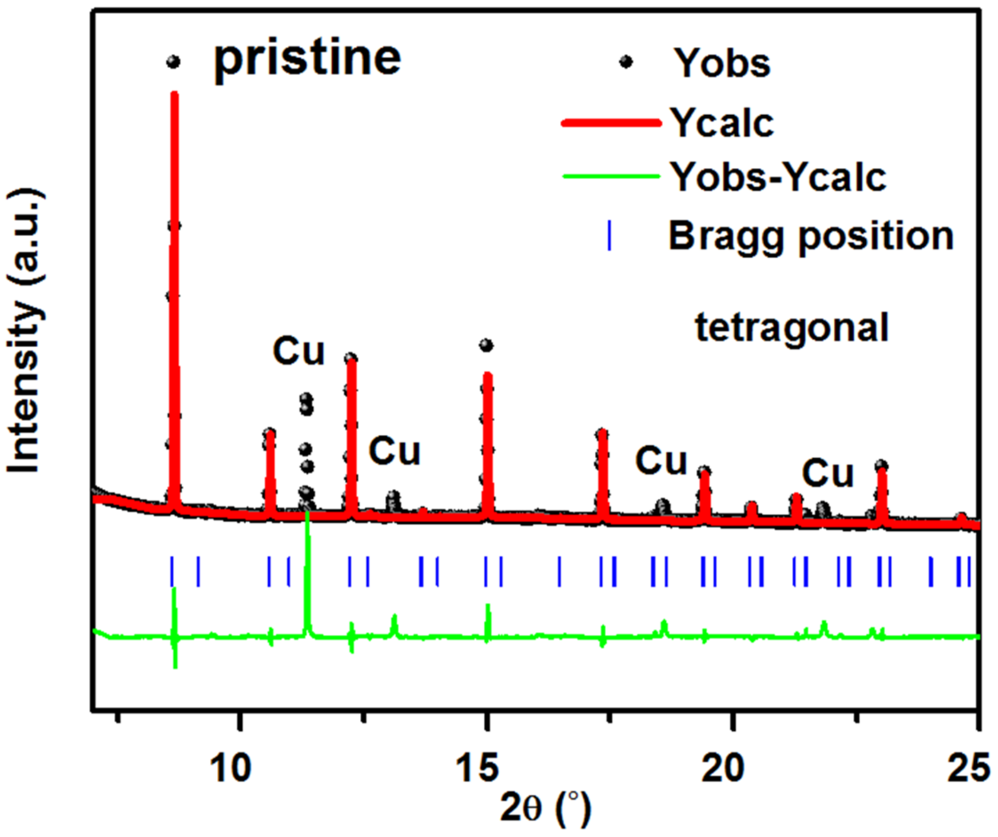

Supplementary Figure 13. The Rietveld refinement of *in situ* synchrotron diffraction of LLTO electrode at initial stage with R_{wp}=4.32% and R_p=2.82%. (tetragonal phase space group: P4/mmm, *a*=*b*=3.87297Å, *c*=7.75497 Å, *V*=116.324Å^3)

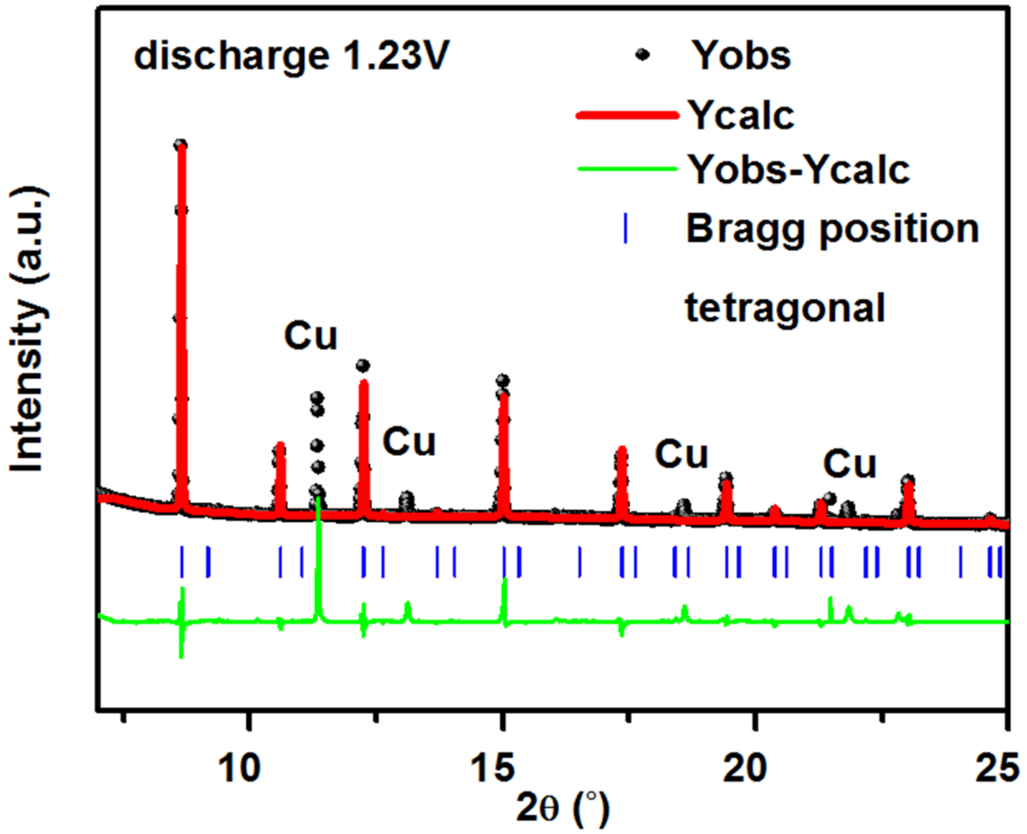

Supplementary Figure 14. The Rietveld refinement of *in situ* synchrotron diffraction of LLTO electrode at discharge to 1.23V with R_{wp}= 6.20% and R_p=3.74%. (tetragonal phase space group: P4/mmm, *a*=*b*=3.87172Å, *c*=7.75227 Å, *V*=116.208Å^3)

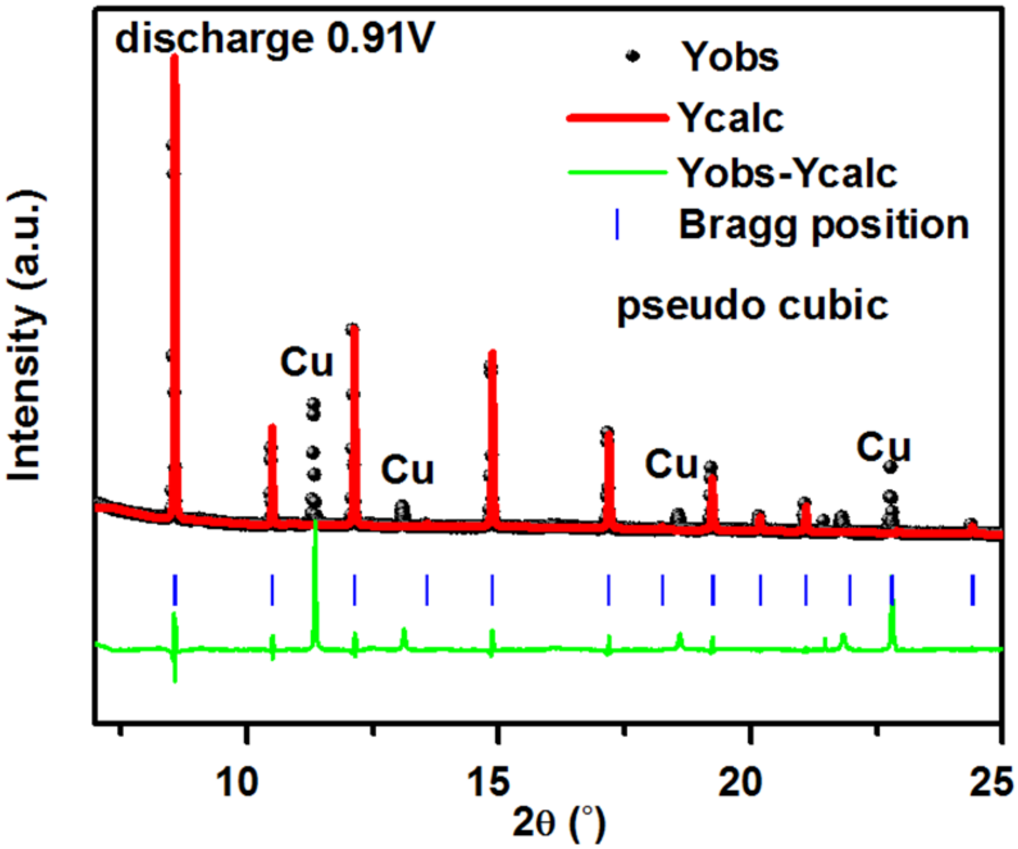

Supplementary Figure 15. The Rietveld refinement of *in situ* synchrotron diffraction of LLTO electrode at discharge to 0.91V with R_{wp}=4.79% and R_p=3.11%. (pseudo-cubic phase space group: P4/mmm, a=b=3.91888 Å, c=3.91537 Å, V=60.131 Å^3)

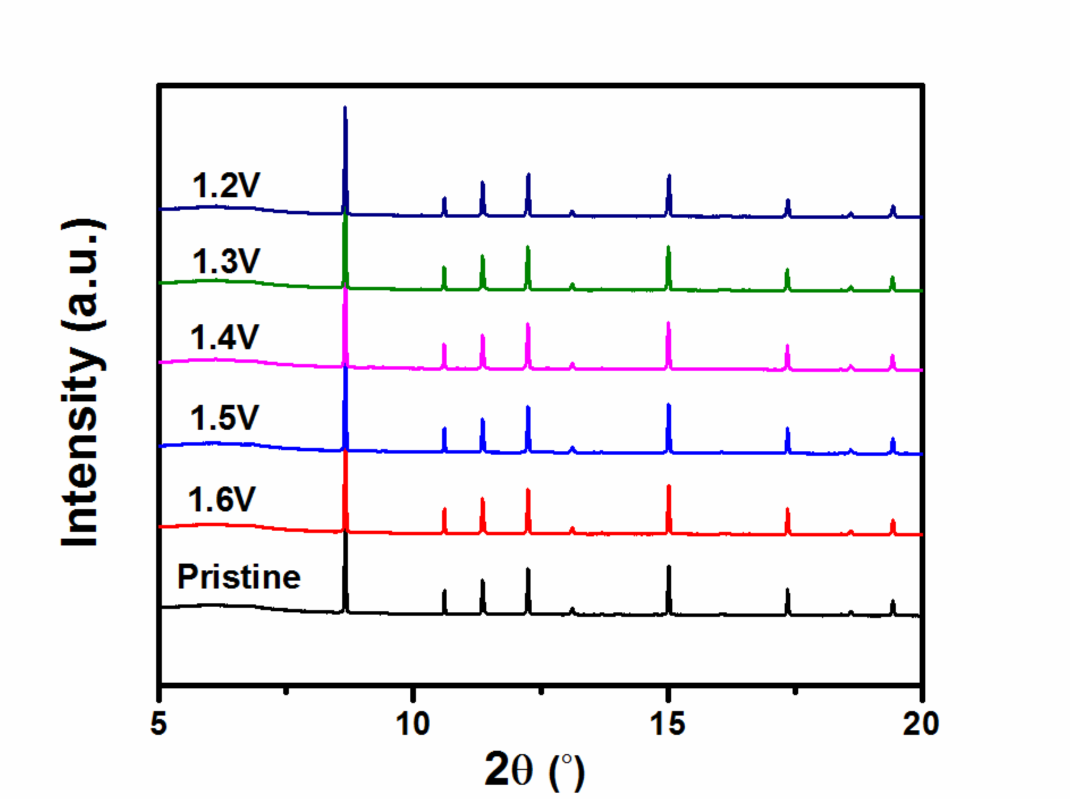

Supplementary Figure 16. The *in situ* synchrotron diffractions of LLTO electrode between pristine and 1.23V.

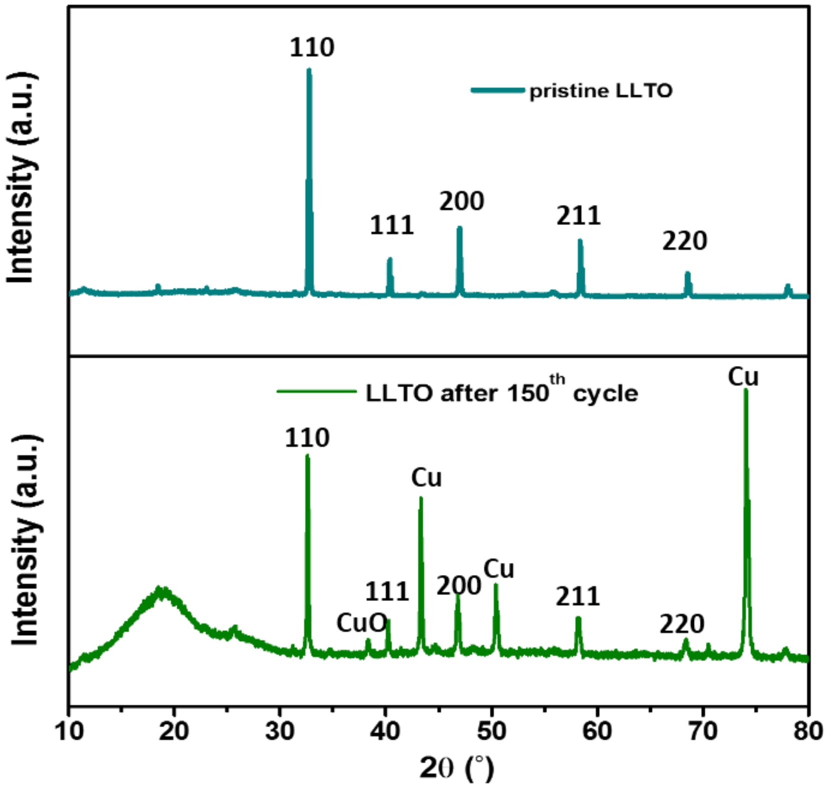

Supplementary Figure 17. XRD of pristine LLTO and *ex situ* XRD of LLTO electrode after 150th cycles. The LLTO electrode maintained original perovskite crystal structure after repeated Li^+ insertion and extraction.

Supplementary Tables

Supplementary Table 1. Rietveld refinement results of LLTO

R_{wp}=7.41%, R_p=5.58%					
P4/mmm a=*b*=3.8811 Å, *c*=7.7591 Å, *α*=*β*=*γ*=90°					
Atom	Wyckoff.	x	y	z	Occupacy
La1	1a	0	0	0	0.041
La2	1b	0	0	0.5	0.032
Li1	1a	0	0	0	0.021
Li2	1b	0	0	0.5	0.031
Ti	2h	0.5	0.5	0.257	0.125
O1	4i	0	0.5	0.236	0.250
O2	1c	0.5	0.5	0	0.062
O3	1d	0.5	0.5	0.5	0.062

Supplementary Table 2. The reported lithium storage properties of the Ti-based oxides.

Materials	Theoretical capacity	Morphology	High rate capacity	Working potential	Reference
TiO_2-Anatase nanosheets	170 mA h g^{-1}	hierarchical spheres assembled by nanosheets less than 1 nm	~150 mA h g^{-1} at 10C	1.70 V	[1]
Micro TiO_2-rutile	336 mAh g^{-1}	Micro particles 5μm	~27 mA h g^{-1} at 5C	1.8V	[2]
TiO_2-B nanopowders	335 mA h g^{-1}	commercial nanopowders	~70 mAh g^{-1} at 10 C	1.70 V	[3]
Micro TiO_2-B	335 mA h g^{-1}	Microrodss 500 nm-1 μm	~80 mAh g^{-1} at 10 C	1.6V	[4]
Micro$Ti_2Nb_{10}O_{29}$	396 mA h g^{-1}	Micro particles 1-5 μm	~168 mAh g^{-1} at 10 C	1.65 V	[5]
Nanoporous $TiNb_2O_7$	387 mA h g^{-1}	Nanoporous sheets 20-200 nm	~220 mAh g^{-1} at 10 C	1.66 V	[6]
Mirco $TiNb_2O_7$	387 mA h g^{-1}	Micro particles 2-10 μm	~100 mAh g^{-1} at 4 C	1.6V	[7]
Carbon coated Porous $Li_4Ti_5O_{12}$	175 mA h g^{-1}	Porous spheres 1-5 μm	~129 mAh g^{-1} at 10 C	1.55 V	[8]
Uncoated Porous $Li_4Ti_5O_{12}$	175 mA h g^{-1}	Porous spheres 1-5 μm	~15 mAh g^{-1} at 10 C	1.55 V	[8]
Nano FeTiO3	355 mA h g^{-1}	Nano particles 200nm-500nm	~56 mAh g^{-1} at 5 C	Under 1.5V	[9]
Micro LLTO	318 mA h g^{-1}	Micro particles 2-20 μm	~100 mAh g^{-1} at 10 C	Under 1 V	This work

Supplementary Table 3. DFT calculated results of LLTO after Li^+ intercalation

Insertion Site	Formation energy (eV)	Internal energy (eV)
A_1 (between La-La atoms)	0.93360968	-295.8480
A_2 (between La-Li atoms)	-0.81239032	-297.5940
A_3 (between Li-Li atoms)	-1.47709032	-298.2587

Supplementary notes

Supplementary note 1. The lithium diffusion coefficient calculation method.

The GITT data are collected at a current rate of 20 mA g^{-1} for 0.5 h and a rest interval of 4 h. The lithium diffusion coefficient can be determined by the following equation:

$$D_{Li} = \frac{4}{\pi\tau}\left(\frac{m_B V_M}{M_B S}\right)^2 \left(\frac{\Delta E_S}{\Delta E_t}\right)^2 \qquad (\tau << L^2/D_{Li}) \qquad (1)$$

where D_{Li} is the lithium-ion diffusion coefficient; τ is the constant current pulse time; m_B is the active material mass; V_M is the molar volume of the material; M_B is the molar mass of the material; S is the active surface area; ΔE_s is the difference between the steady potentials; and ΔE_t is the total transient voltage change of the cell for an applied galvanostatic current for the time τ.[10]

Supplementary References

[1] Chen, J., Tan, Y., Li, C., Cheah, Y., Luan, D., Madhavi, S., Boey, F., Archer, A., & Lou, X. Constructing Hierarchical Spheres from Large Ultrathin Anatase TiO_2 Nanosheets with Nearly 100% Exposed (001) Facets for Fast Reversible Lithium Storage. *J. Am. Chem. Soc.* **132**, 6124–6130 (2010).

[2] Gardecka, A., Lübkea, M., Armerb, C., Ning, D., Reddy, M., Williams, A., Lowe, A., Liu, Z., Parkin, I., & Darr, J., Nb-doped rutile titanium dioxide nanorods for lithium-ion batteries. *Solid State Sci*. **83**, 115–121(2018).

[3] Liu, H., Bi, Z., Sun, X.-G., Unocic, R. R., Paranthaman, M. P., Dai, S. & Brown, G. M., Mesoporous TiO_2–B Microspheres with Superior Rate Performance for Lithium Ion Batteries. *Adv. Mater.* **23**, 3450–3454 (2011).

[4] Nakano, Y., Masuda,K., Takagi, M., Saito, M., Tasaka, A., & Inaba, M. Improvement of Tap Density of TiO_2(B) Powder as High Potential Negative Electrode. ECS Transactions, **50** (26), 261-269 (2013).

[5] Cheng, Q., Liang, J., Zhu, Y., Si, L., Guo, C. & Qian, Y. Bulk $Ti_2Nb_{10}O_{29}$ as long-life and high-power Li-ion battery anodes. *J. Mater. Chem. A*, **2**, 17258–17262 (2014).

[6] Guo, B., Yu, X., Sun, X., Chi, M., Qiao, Z., Liu, J., Hu, Y., Yang, X., Goodenough, J. & Dai, S. A long-life lithium-ion battery with a highly porous $TiNb_2O_7$ anode for large-scale electrical energy storage. *Energy Environ. Sci.* **7**, 2220–2226 (2014).

[7] Han, J., Huang, Y., & Goodenough, J. New Anode Framework for Rechargeable Lithium Batteries *Chem. Mater.* **23**, 2027–2029 (2011).

[8] Zhao, L., Hu. Y., Li. H., Wang, Z. & Chen. L., Porous $Li_4Ti_5O_{12}$ Coated with N-Doped Carbon from Ionic Liquids for Li-Ion Batteries. *Adv. Mater.* **23**, 1385–1388 (2011).

[9] Tao, T., Glushenkov, A., Rahman, M., Chen, Y., Electrochemical reactivity of ilmenite $FeTiO_3$, its nanostructures andoxide-carbon nanocomposites with lithium *Electrochimica Acta* **108**. 127–134 (2013).

[10] Li, Z., Du, F., Bie, X., Zhang, D., Cai,Y., Cui, X., Wang, C., Chen, G., & Wei, Y. Electrochemical Kinetics of the $Li[Li_{0.23}Co_{0.3}Mn_{0.47}]O_2$ Cathode Material Studied by GITT and EIS. *J. Phys. Chem. C* **114**, 22751–22757(2010).
